# Fabrication of Lead-Free Bi_0.5_Na_0.5_TiO_3_ Thin Films by Aqueous Chemical Solution Deposition

**DOI:** 10.3390/ma10020213

**Published:** 2017-02-22

**Authors:** Mads Christensen, Mari-Ann Einarsrud, Tor Grande

**Affiliations:** Department of Materials Science and Engineering, NTNU Norwegian University of Science and Technology, Trondheim NO-7491, Norway; mads.j.christensen@ntnu.no (M.C.); mari-ann.einarsrud@ntnu.no (M.-A.E.)

**Keywords:** Bi_0.5_Na_0.5_TiO_3_, bismuth sodium titanate, BNT, NBT, aqueous, sol-gel, chemical solution deposition (CSD), lead-free, thin-film, piezoelectric, phase purity

## Abstract

Piezoelectric ceramics are widely used in actuator applications, and currently the vast majority of these devices are based on $\mathrm{Pb}\left(\mathrm{Zr},\mathrm{Ti}\right)O_{3}$, which constitutes environmental and health hazards due to the toxicity of lead. One of the most promising lead-free material systems for actuators is based on ${\mathrm{Bi}}_{0.5}{\mathrm{Na}}_{0.5}{\mathrm{TiO}}_{3}$ (BNT), and here we report on successful fabrication of BNT thin films by aqueous chemical solution deposition. The precursor solution used in the synthesis is based on bismuth citrate stabilized by ethanolamine, NaOH, and a Ti-citrate prepared from titanium tetraisopropoxide and citric acid. BNT thin films were deposited on ${\mathrm{SrTiO}}_{3}$ and platinized silicon substrates by spin-coating, and the films were pyrolized and annealed by rapid thermal processing. The BNT perovskite phase formed after calcination at 500 °C in air. The deposited thin films were single phase according to X-ray diffraction, and the microstructures of the films shown by electron microscopy were homogeneous and dense. Decomposition of the gel was thoroughly investigated, and the conditions resulting in phase pure materials were identified. This new aqueous deposition route is low cost, robust, and suitable for development of BNT based thin film for actuator applications.

## 1. Introduction

Piezoelectric ceramics are widely used in actuator micro electro-mechanical systems (MEMS) such as fuel injection dies and precision positioning devices. The vast majority of these devices are based on $\mathrm{Pb}\left(\mathrm{Zr},\mathrm{Ti}\right)O_{3}$ (PZT) [1], which constitutes environmental and health hazards due to the toxicity of lead [2]. For this reason, considerable research efforts have focused on developing alternative lead-free piezoelectric materials [3,4,5,6,7]. One of the most promising lead-free materials for actuators is based on ${\mathrm{Bi}}_{0.5}{\mathrm{Na}}_{0.5}{\mathrm{TiO}}_{3}$ (BNT) substituted with BaTiO_3_ (BT), due to a large strain response originating from a reversible electric field induced relaxor to ferroelectric phase transition, observed at the morphotropic phase boundary at 6 mol % ${\mathrm{BaTiO}}_{3}$ (BNT-6BT) [4,8]. The exact nature of the phase transitions in the BNT-BT system below $T_{C}$ is still a matter of debate. A detailed review of the properties of BNT-based materials with regard to actuator applications has recently been reported [9].

Chemical solution deposition (CSD) is a low cost and versatile method to achieve high quality and homogeneous thin films with good stoichiometry control [10]. PZT and other oxide thin films are routinely prepared via CSD [11]. Precursor chemistries based on 2-methoxyethanol (2-MOE) or other organic solvents have achieved the role of a standard which other CSD methods are often compared to [12,13]. Synthesis routes employing 2-MOE have been developed for, among others, PZT [12], $K_{1-x}{\mathrm{Na}}_{x}{\mathrm{NbO}}_{3}$ (KNN) [14] and ${\mathrm{BaTiO}}_{3}$ (BT) [15], as well as for BNT [16,17,18,19,20,21,22]. However, 2-MOE and other organic solvents are both toxic and expensive, and aqueous thin film processing is therefore desirable.

Reports on aqueous chemical solution synthesis of BNT is scarce in the literature, and to our knowledge, aqueous thin film deposition has yet to be demonstrated. West et al. have reported to stabilize Ti and Na cations in solution by citric acid, while the chemistry of dissolving $\mathrm{Bi}{\left(\mathrm{OH}\right)}_{3}$ was not properly accounted for [23]. In this work, it was assumed that stabilization of the Bi ions in the solution was related to a combined Bi-Na citrate complex. Furthermore, the stability of the sol, with a concentration of less than 0.2 M, was not reported. Xu et al. prepared a Ti-citrate solution from tetrabutyl titanate and citric acid, into which ${\text{NH}}_{4}\text{OH}$, ${\mathrm{NaNO}}_{3}$, $\mathrm{Ba}{\left({\mathrm{NO}}_{3}\right)}_{2}$, and $\mathrm{Bi}{\left({\mathrm{NO}}_{3}\right)}_{3}\cdot {5H}_{2}O$ were added [24,25,26,27]. A total citric acid to cation concentration ratio of 1.2–1.6 yielded a transparent homogeneous sol with pH 6, but the stability timeframe of the sol was not addressed. Aqueous synthesis of other related materials introduces different stabilization techniques for the constituent cations of BNT. ${\mathrm{Bi}}_{4}{\mathrm{Ti}}_{3}O_{12}$ has been synthesized from Bi-citrate by dissolving with ${\text{NH}}_{4}\text{OH}$ and stabilizing with ethanolamine [28], and ${\mathrm{TiO}}_{2}$ has been synthesized by stabilizing Ti-isopropoxide by citric acid [29].

Here, we report on aqueous chemical solution deposition of BNT thin films. Two different sol systems were investigated. The first, referred to as the citrate sol, employed bismuth citrate stabilized by ethanolamine [28] and NaOH. In the second sol, the nitrate sol, $\mathrm{Bi}{\left({\mathrm{NO}}_{3}\right)}_{3}\cdot {5H}_{2}O$ and ${\mathrm{NaNO}}_{3}$ were used, similar to the method of Xu et al. [25]. Ti-isopropoxide stabilized by citric acid [29] was used as Ti precursor. The sols were compared with respect to properties of the resultant oxide, and the feasibility of employing the synthesis route in production lines. Thin films were fabricated by spin-coating the sol onto single crystalline ${\mathrm{SrTiO}}_{3}$ and platinized silicon substrates, and the thermal processing parameters governing phase purity were thoroughly investigated. Finally, BNT powders were also prepared by the two routes, providing supplementary insight into the decomposition and crystallization processes. The main focus was devoted to the citrate route, which proved to be more successful. The findings of the nitrate method are presented in Appendix A.

## 2. Results

### 2.1. Deposition of Thin Film Using the Citrate Sol

**Figure 1:**
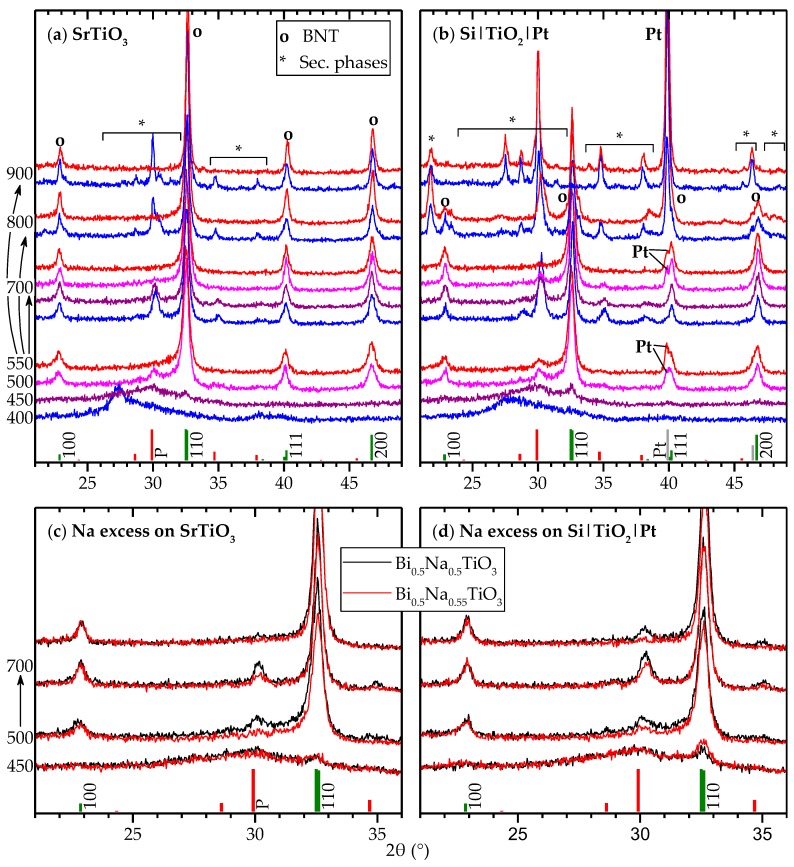
Grazing incidence X-ray diffraction (XRD) patterns of deposited ${\mathrm{Bi}}_{0.5}{\mathrm{Na}}_{0.5}{\mathrm{TiO}}_{3}$ (BNT) films: (**a**) films deposited on SrTiO_3_ (STO) substrates; (**b**) films deposited on platinized silicon (SiPt) substrates; (**c**) comparison of nominal stoichiometric (black) and 10% Na excess (red) sols on STO; and (**d**) comparison of stoichiometric and Na excess on platinized silicon. Heat treatment temperatures are denoted to the left. Arrows indicate that samples were first pyrolized for each layer, then finally annealed at the given temperature. Color codes correspond to the pyrolysis temperatures. Reference patterns are shown for R3c BNT (pseudo cubic hkl indices) [30], a Na-poor pyrochlore phase (“P”) [31] and platinum (“Pt”).

BNT thin films were successfully deposited on both types of substrates, and grazing incidence X-ray diffraction (XRD) of the films are displayed in Figure 1. Phase pure BNT perovskite was achieved on ${\mathrm{SrTiO}}_{3}$ (STO) substrates (Figure 1a) after pyrolysis at 550 °C, or pyrolysis at 500 °C followed by thermal processing at 700 °C. The films deposited on platinized silicon (SiPt) (Figure 1b), were phase pure after pyrolysis at 550 °C and sintering at 700 °C, thus requiring higher thermal processing temperature than STO films. The unit cell parameters and the crystallite size obtained by Rietveld refinement for the films are summarized in Table A1, and crystallographic data are in reasonable agreement with literature [30].

The pyrolysis temperature was demonstrated to be critical with respect to the phase purity of the films. The ratio of BNT to secondary phases obtained during pyrolysis was retained after annealing, irrespectively of further thermal processing. STO films pyrolized at 550 °C remains single phase BNT after thermal annealing, while the amount of secondary phases in thin films pyrolized at 400 °C was not altered after thermal annealing at 700, 800 and 900 °C. The diffraction patterns of the secondary phase is reasonably well described by a pyrochlore phase [31], although the chemical composition of the pyrochlore was affected by the thermal processing, as evidenced by the shift in the position of the main diffraction lines.

Black color of the films and broad Bragg reflections, observed after pyrolysis at 400 °C, is proposed to originate from the formation of metallic bismuth. The formation of elemental Bi results in secondary phases upon re-oxidation during further thermal processing of the films. Several bismuth rich structures, such as ${\mathrm{Na}}_{0.32}{\mathrm{Bi}}_{1.68}{\mathrm{Ti}}_{2}O_{6.46}{\left(\mathrm{OH}\right)}_{0.44}$ [31] and ${\mathrm{Bi}}_{4}{\mathrm{Ti}}_{3}O_{12}$ [32], match the observed secondary phase well, suggesting that these phases grew from areas enriched in bismuth.

Thin films deposited on STO and SiPt substrates behaved similarly for heat treatments up to 700 °C, but a range of secondary phases appeared at 800–900 °C in the thin films on SiPt (Figure 1b). These films turned matte grey in color and appeared inhomogeneous to the naked eye. Additionally, at 900 °C, BNT formed during pyrolysis disappeared, reflecting the strong change in the visible appearance. Note also that Pt reflections became apparent for samples pyrolized at 500–550 °C, but not for samples pyrolized at 400–450 °C even after annealing at 700 °C, indicating that there is some degree of Pt recrystallization during pyrolysis, but not during further thermal processing. Platinum bottom electrodes are the industry standard for electronic oxide thin films, and compatibility towards Pt is essential. For SiPt substrates, pyrolysis at 550 °C and annealing at 700 °C yielded phase pure samples.

Thin films prepared from a sol containing 10% sodium excess clearly reduced the amount of pyrochlore at any given heat treatment for films on both STO and SiPt substrates (Figure 1c,d).

**Figure 2:**
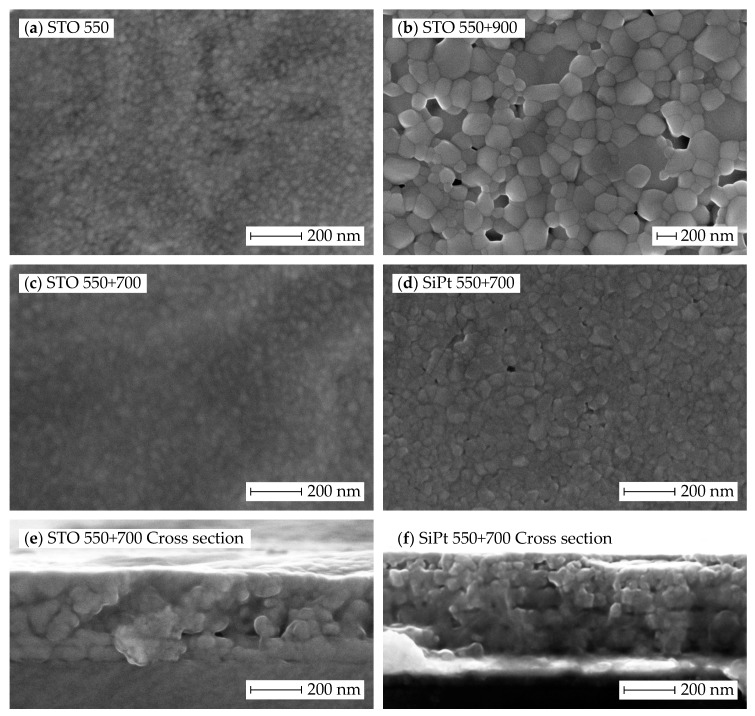
Secondary electron scanning electron microscope (SEM) images of phase pure BNT films prepared by the citrate sol: (**a**) top view of films on STO pyrolized at 550 °C before; and (**b**) after sintering at 900 °C; (**c**) top; and (**e**) cross section view of films on STO; and (**d**,**f**) SiPt pyrolized at 550 °C and sintered at 700 °C.

Representative thin films from the heat treatment procedures yielding phase pure films were selected for investigation by scanning electron microscope (SEM). Figure 2 shows that the films were homogenous and fairly dense, and that the thickness of the films is approximately 300 nm for five layers. At the surface (5th layer), the grain size is approximately 20 nm in the thin films on STO, both before and after annealing at 700 °C, while in case of SiPt substrates, the grain size is slightly larger. Cross section images show that the grain size is larger in the interior of the film than at the surface. Sintering at 900 °C yields significantly larger grains on STO, approximately 200 nm.

### 2.2. Decomposition of Gel from Citrate Sol

**Figure 3:**
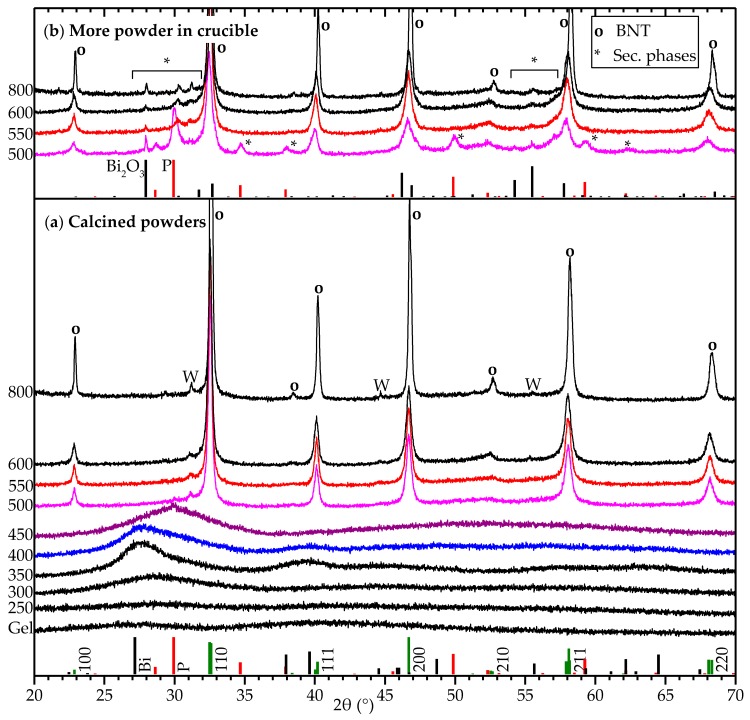
(**a**) XRD patterns of BNT powders calcined for 2 h in ambient atmosphere. The temperature interval 400–550 °C is color coded to augment comparisons between figures. Reference patterns are shown for R3c BNT (pseudo cubic hkl indices) [30], metallic bismuth (“Bi”), a Na-deficient pyrochlore phase (“P”) [31] and ${\mathrm{Bi}}_{2}O_{3}$. Reflections denoted by W are caused by tungsten Lα1 radiation from the instrument. Rietveld refinement of the patterns are presented in Table A1, showing reasonable agreement with literature data [30]; (**b**) Powders calcined simultaneously as in (**a**), but with larger amounts of powder in the crucibles.

Supplementary to thin films, powder samples were prepared by drying a portion of the sols used for deposition of thin films. XRD patterns of powders heat treated at various temperatures in ambient atmosphere are shown in Figure 3a. From 500 to 800 °C, the samples were phase pure BNT according to XRD. The crystallinity of the powders increased by increasing the calcination temperature, and the reflection at about 52°, as well as the rhombohedral super-reflection at 38°, became more pronounced.

Heat treatment up to 250 °C yielded amorphous samples, showing that evaporation of the solvent did not lead to crystallization or precipitation, suggesting that the dried gel is homogeneous down to the atomic level. After heat treatment at 300 to 400 °C, however, broad reflections corresponding to metallic bismuth appeared, and these samples were black. The bismuth particles were nano-sized, increasing in size with temperature, as indicated by the width of the reflections.

Access to oxygen was essential for obtaining phase pure samples. Figure 3b displays XRD patterns of samples prepared together with the samples in Figure 3a, but with significantly more powder in the crucibles. After calcination, these samples were inhomogeneous, as the outer layers were white, while the cores were yellow or black, and XRD confirmed the presence of secondary phases. This can be explained by the reduction of bismuth during heating, and slower bismuth re-oxidation where oxygen access is limited, allowing for Ostwald ripening and segregation of Bi and subsequent formation of secondary phases upon re-oxidation of Bi.

**Figure 4:**
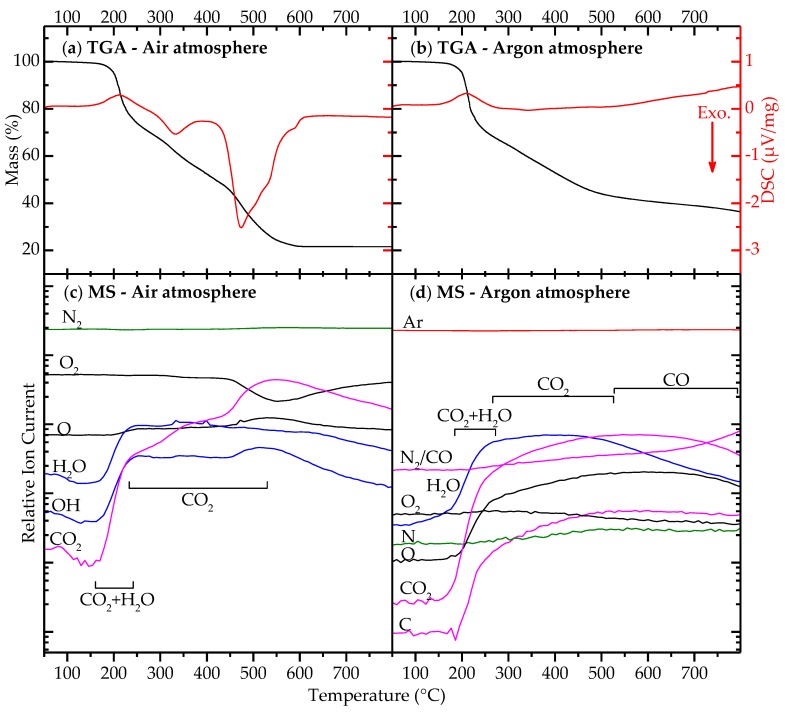
Thermogravimetric analysis (TGA) of dried BNT gel: (**a**,**b**) mass loss (black, left axis) and differential scanning calorimetry (red, right axis); and (**c**,**d**) in situ mass spectroscopy (MS) of TGA outlet gas. Different graphs correspond to specific *m*/*z* values, labeled with the gaseous species contributing to the *m*/*z* current. Lines are color coded: green, nitrogen; black, oxygen; blue, water; magenta, carbon; and red, argon. Dominating gaseous species for mass loss are indicated next to MS-curves. In (**d**), combined assessment of curves for N, C and ${\mathrm{CO}}_{2}$ shows that $N_{2}$/CO signal originates from $N_{2}$ at low temperature and CO at high. Ramp up rate: 10 °C/min.

Thermogravimetric analysis with outlet gas mass spectroscopy (TGA-MS) of the gel during heating is shown in Figure 4. Evaporative loss of water (endothermic) and ${\mathrm{CO}}_{2}$ occurred at 200 °C. Decomposition of the gel and formation of metallic bismuth corresponds to an exothermic mass loss at 300 °C. Accompanied by a significant consumption of oxygen, several exothermic events overlapped at 450–550 °C, one of which was likely related to bismuth re-oxidation. The mass loss stabilized at 79% at approximately 550 °C, and the sample recovered from the TGA experiment was phase pure BNT according to XRD (not shown). Figure 4b,d shows that the initial release of water and ${\mathrm{CO}}_{2}$ was independent of oxygen access, but increasing the temperature further in argon atmosphere led to incomplete decomposition, and none of the exothermic events observed in air occurred. At 800 °C, weight loss was still incomplete, and the recovered sample contained no perovskite phase, but primarily metallic bismuth according to XRD (not shown).

**Figure 5:**
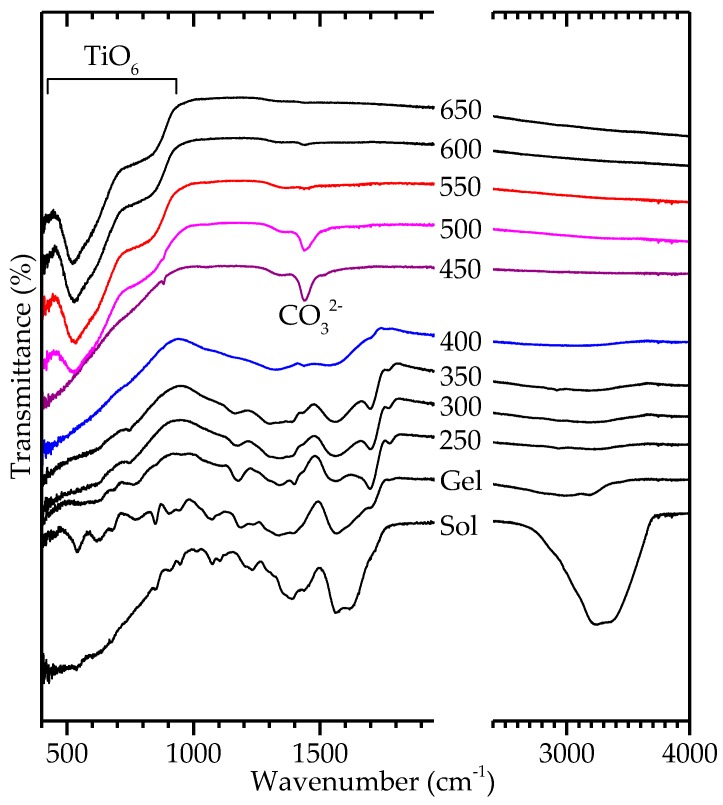
Fourier transform infrared (FTIR) spectra of calcined citrate BNT powders. The scale is changed at wavenumber 2000 cm^−1^ in order to more clearly show the features of interest. Consecutive graphs are offset the same value.

IR transmittance spectra of the powders calcined at different temperatures are shown in Figure 5. In accordance with XRD and TGA experiments, decomposition of the gel was complete after calcination at 550 °C, leaving only bands related to the ${\mathrm{TiO}}_{6}$ octahedra in BNT. The band at 1430 cm^−1^ corresponds to a carbonate phase formed by the decomposition of the organics [33] (p. 284).

## 3. Discussion

To the best of our knowledge, this is the first report on preparation of BNT thin films by an aqueous chemical solution deposition method. The citrate method reported here resulted in single phase, homogeneous and dense BNT thin films. Furthermore, the preparation of the sol does not require any costly, time consuming, or complicated steps, and results in a stable, pH neutral sol free of harmful or environmentally toxic chemicals. Due to the simple synthesis and robust method, the results obtained are easily reproducible and are suitable for further modification, such as addition of Ba or up-scaling.

**Figure 6:**
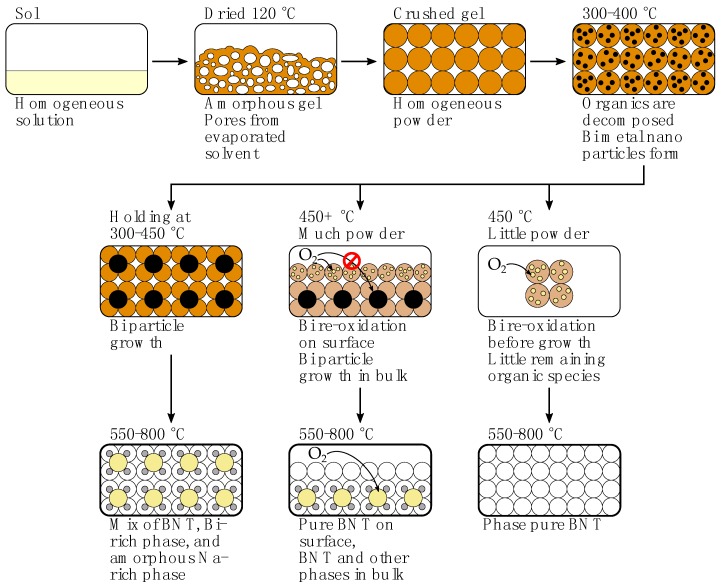
Proposed decomposition process for citrate method powders. In the bottom panels, yellow signifies a bismuth rich secondary phase (e.g., ${\mathrm{Bi}}_{2}O_{3}$), grey signifies an amorphous sodium rich secondary phase invisible to XRD, and white signifies BNT.

The process of decomposition of the gel into oxide powders and thin films was studied in detail. Reduction of ${\mathrm{Bi}}^{3+}$ and the growth and segregation of metallic Bi particles is the main challenge of the synthesis, potentially leading to secondary phases during re-oxidation of bismuth. A hypothesis for the decomposition process in powder samples is presented in Figure 6, where the various parameters controlling phase purity of the product are highlighted. During drying, the viscosity of the sol increases gradually as water and volatile species evaporate, leading to a brittle and porous gel, which is amorphous and homogeneous on the atomic level. Upon heating past 300 °C, the hybrid inorganic-organic gel decomposes, causing oxidation of organic constituents and reduction of ${\mathrm{Bi}}^{3+}$. ${\mathrm{Bi}}^{3+}$ has previously been shown to act as an efficient oxidizing agent for benzylic, allylic and aliphatic alcohols [34]. Initially, metallic bismuth takes the form of nano-sized liquid droplets, and appears as nano-crystalline Bi in XRD patterns after cooling to ambient temperature. During further annealing at 300–450 °C, however, the bismuth droplets grow through Ostwald ripening, causing increasing levels of bismuth segregation and formation of phase pure BNT is hindered due to the segregation of Bi. The result is therefore bismuth rich secondary phases containing a varying amount of titanium and sodium, such as ${\mathrm{Bi}}_{2}O_{3}$ and pyrochlore (${\mathrm{Na}}_{0.32}{\mathrm{Bi}}_{1.68}{\mathrm{Ti}}_{2}O_{6.46}{\left(\mathrm{OH}\right)}_{0.44}$) [31]. If, however, the temperature during calcination is high enough to allow re-oxidation of Bi and crystallization of BNT perovskite, the growth of bismuth droplets and segregation is hindered, yielding phase pure BNT. The temperature limit for these two scenarios was shown to be 450–500 °C.

Even if the temperature is high enough for bismuth re-oxidation, access to oxygen may still be limiting. If the layer of powder in the crucible is too thick, re-oxidation will be diffusion controlled, and Bi growth will be allowed to occur in the core of the powder bed, again resulting in secondary phases. We therefore can conclude that in order to obtain phase pure powders, calcination should be carried out at 450–500 °C or higher, and sufficient oxygen should be present.

**Figure 7:**
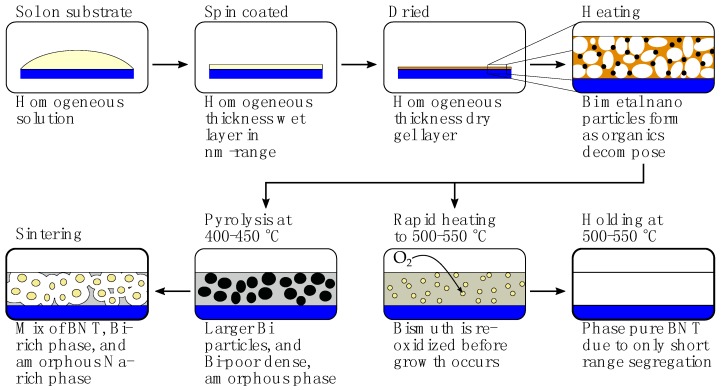
A schematic of the decomposition process of citrate method thin film samples. The substrate is drawn blue, pores and BNT are white, gel is brown, Bi rich secondary phase is yellow, and an amorphous Na rich secondary phase is drawn grey.

The thermal processing of thin films encountered the same challenges as observed in the preparation of powders. Metallic bismuth was shown to be formed during pyrolysis at 400 °C, and a pyrochlore secondary phase was observed for several heat treatment routes. A schematic for the proposed processes involved during thermal processing of the thin films, and the influence of heat treatment parameters is presented in Figure 7. First, the sol is spin-coated onto the substrate and dried, giving a thin homogeneous layer of the gel. As shown for the powder, decomposition and oxidation of the hybrid gel is accompanied with reduction of ${\mathrm{Bi}}^{3+}$, forming metallic bismuth. Observing that formation of metallic Bi occurred in case of both powder and thin film samples, where heating rates, holding times, and diffusion lengths are significantly different, the reduction is concluded to be a result of the local environment of Bi in the gel, and not the access to oxygen during decomposition of the gel. Thus, sufficiently high pyrolysis temperature is essential, as pyrolysis at 400–450 °C promotes Ostwald ripening of Bi(l), while pyrolysis at 500–550 °C cause rapid Bi re-oxidation, hindering segregation. It is concluded that the final phase purity is largely determined by the pyrolysis temperature. Any segregation and secondary phases formed during pyrolysis will remain after further thermal processing, independent of the thermal processing temperature. Pyrolysis of the BNT thin films at 500 °C or higher was demonstrated to be key to obtain phase pure BNT thin films.

Reduction of bismuth during chemical solution processing of oxides is known, but rarely reported [34]. For example, Nelis et al. prepared ${\mathrm{SrBi}}_{2}{\mathrm{Nb}}_{2}O_{9}$ by a similar method and report metallic bismuth after heat treatment at 400 °C [35]. Furthermore, the connection between segregation during decomposition and resultant secondary phases after subsequent heat treatment is frequently not recognized. Often, in related material systems, secondary phases are simply accredited to evaporation of volatile species, while it is likely that bismuth reduction and segregation during pyrolysis is equally important. For example, Xu et al. report a ${\mathrm{Bi}}_{4}{\mathrm{Ti}}_{3}O_{12}$ secondary phase (similar to the pyrochlore reported here) after heat treatment at 500–550 °C when preparing BNT-BT [25,26]. Li et al. made BNT-BKT-BZT (K and Zn doped BNT) thin films by an organic solvent procedure, and reported a small amount of pyrochlore after pyrolysis at 400 °C and sintering at 700 °C [36]. Alonso-Sanjosé et al. also made BNT-BT thin films by organic solvent sol-gel method, and reported formation of a pyrochlore phase after heat treatment at 600 °C, but not after 650 °C [37]. Moreover, Zhou et al. prepared BNT-BT films, and observed that pyrolysis at 350 °C resulted in the presence of a pyrochlore phase after sintering at 700 °C, while pyrolysis at 400 °C did not [17]. In the latter case the authors presented a possible explanation which resembles the one presented here.

Sodium excess was shown to efficiently reduce the amount of pyrochlore secondary phase for all the heat treatment procedures used in this work. Both bismuth and sodium are known to be volatile, and adding excess Bi- or Na precursors during synthesis is common. It has been shown that the BNT material system cannot accommodate a large amount of A to B non-stoichiometry, and less than 1 mol % variation cause formation of secondary phases (i.e., ${\mathrm{Bi}}_{2}O_{3}$ for Bi rich, ${\mathrm{Na}}_{2}{\mathrm{Ti}}_{6}O_{13}$ for Na rich, and ${\mathrm{TiO}}_{2}$ for Ti rich) [38,39]. However, increasing the Na to Bi ratio acts as A site acceptor doping, which is charge compensated by oxygen vacancies, leading to a conductivity increase by several orders of magnitude [38,39]. Additionally, Na excess cause hardening of BNT-BT ferroelectrics [40]. Therefore, while sodium excess is efficient in reducing the amount of pyrochlore phase, care should be taken as it might significantly reduce the piezoelectric properties. A possible remedy for the negative effects of Na excess is addition of some donor dopant, such as ${\mathrm{Nb}}^{5+}$ on B site, leaving sodium excess samples still relevant for final application.

When films deposited on platinized silicon were heat treated above 700 °C, a range of secondary phases evolved due to a reaction between BNT and the platinum bottom electrode. Alonso-Sanjosé et al. report formation of a Bi-Pt secondary phase when heat treating BNT thin films on platinized silicon at 750 °C [37]. Considering the high solubility in the Bi-Pt binary system, this reaction is a likely explanation. For example, a 50-50 at % Bi-Pt alloy melts at 765 °C, and the melting point only decreases for higher Bi content [41]. For implementation of BNT thin films on silicon wafers, where Pt has achieved the role as the standard bottom electrode, either suppressing the Pt-Bi reaction, or replacing Pt in favor of another electrode may be necessary.

Changes in the Pt bottom electrode were also observed at lower heat treatment temperatures. XRD patterns recorded in grazing incidence mode for clean substrates or samples pyrolized at 400–450 °C (Figure 4b) did not show Pt reflections, but Pt reflections became visible after pyrolysis at 500–550 °C. The same trends have also been reported for ${\mathrm{Bi}}_{3.25}{\mathrm{Nd}}_{0.75}{\mathrm{TiO}}_{12}$ on platinized silicon, showing Pt reflections after annealing at 500–650 °C, but not after annealing at 400 °C [42]. Initially, the Pt layer is textured with (111) orientation, and in grazing incidence mode horizontally oriented planes will not appear. Appearance of Pt reflections can be explained by recrystallization of the Pt layer, which could be induced by impurities, most likely Bi diffusion from the deposited film. We suggest that the absence of recrystallization at 400–450 °C is due to lower mobility and solubility in the Bi-Pt system at low temperature. At higher temperatures, a small amount of Bi-Pt interdiffusion takes place, causing Pt recrystallization. However, when the stable perovskite phase forms, the reactivity of Bi is reduced, explaining why no further Pt recrystallization is observed during annealing at 700 °C.

Processing of thin films was the focus of the current work, and for that reason, obtaining phase pure powders in large quantities was not investigated. However, there are clear advantages to using a chemical solution method when producing powders, such as small particle size, excellent control of stoichiometry and low heat treatment temperatures. Solid state synthesis of BNT typically requires sintering temperatures in excess of 1100 °C [43], where volatility of bismuth and sodium can give poor stoichiometry control, and where significant grain growth is difficult to avoid. Compared to the other reports on aqueous BNT processing, the citrate method developed here shows advantages such as high stability and pH neutrality of the sol, cheap precursors and a simple process. For bulk powder production, a sufficient flow of oxygen for re-oxidation of Bi is necessary.

The most attractive piezoelectric properties of BNT based materials are found for materials with barium A site doping as well as a small amount of B site doping such as Nb or Zr [44]. Due to the robustness, low cost, and benign characteristics of the citrate sol, this method is well suited for further modifications such as barium doping, making the samples relevant for actuating applications.

## 4. Materials and Methods

### 4.1. Sol Preparation

**Figure 8:**
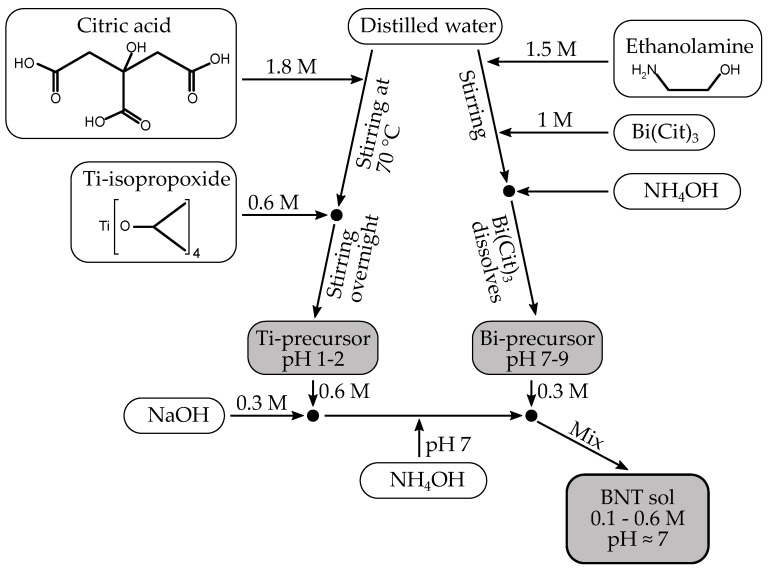
Flow-chart of the citrate method for BNT sol preparation.

Two different recipes to BNT sols were investigated. Both recipes used Ti(IV)-isopropoxide (>97%, Sigma-Aldrich, Darmstadt, Germany, SKU 87560) stabilized by citric acid (99%, Sigma-Aldrich, SKU C0759) as titanium precursor [29]. In the first recipe, referred to as citrate method, the other precursors were Bi-citrate (99.99%, Sigma-Aldrich, SKU 480746) stabilized by ethanolamine (>99.5%, Sigma-Aldrich, SKU 411000) [28], NaOH solution (4.00 M, Sigma-Aldrich, SKU 35274), and finally ${\text{NH}}_{4}\text{OH}$ solution (33%, Sigma-Aldrich, SKU 05002) was used in order to increase pH of the final sol. The second recipe, the nitrate method, used $\mathrm{Bi}{\left({\mathrm{NO}}_{3}\right)}_{3}\cdot {5H}_{2}O$ (>98%, Sigma-Aldrich, SKU 383074), ${\mathrm{NaNO}}_{3}$ (>99%, VWR, Radnor, PA, USA, Cnr 14493), polyvinyl alcohol (PVA, Sigma-Aldrich, SKU 341584) as surfactant, and finally ${\mathrm{HNO}}_{3}$ (65%, Merck KGaA, Darmstadt, Germany, Pnr 1.00456) in order to decrease pH. In both cases, distilled water was used as solvent, yielding sols of varying concentration, from 0.1 M to 0.6 M. A flow-chart illustrating the preparation of citrate sol is shown in Figure 8.

The Ti precursor was prepared by heating 1.8 M citric acid in distilled water to 80 °C, followed by addition of Ti-isopropoxide via a syringe to 0.6 M final concentration. Upon addition of Ti-isopropoxide, a precipitate was immediately formed yielding a white opaque slurry. After stirring at 70 °C overnight, the solution turned clear with a yellow tint, signifying the formation of a $\mathrm{Ti}{\left(\mathrm{cit}\right)}_{3}$ complex as described by Deng et al. [45]. Concentration was adjusted to approximately 0.6 M in Ti by diluting with distilled water or careful evaporation at 70 °C depending on the volume of the solution. Finally, the exact concentration was measured by heat treatment of three parallels of 1.00 mL solution at 1000 °C for 8 h, and measuring the mass of resultant ${\mathrm{TiO}}_{2}$. The precursor had pH equal to 1, and showed no sign of ageing or precipitation even after 10 months.

For the citrate method, Bi-ethanolamine complex was formed by stirring Bi-citrate and ethanolamine in a 1:1.5 molar ratio in distilled water. In order to dissolve Bi-citrate, ${\text{NH}}_{4}\text{OH}$ was added to pH = 8–9, and a clear 1.0 M solution was obtained. The complete BNT sol was finally prepared by adding, in molar ratio, 1 part Ti precursor, 0.5 parts NaOH, 2 parts ${\text{NH}}_{4}\text{OH}$, and 0.5 parts Bi precursor, in chronological order.

The nitrate sol was prepared by adding ${\mathrm{NaNO}}_{3}$, $\mathrm{Bi}{\left({\mathrm{NO}}_{3}\right)}_{3}\cdot {5H}_{2}O$, ${\mathrm{HNO}}_{3}$, and a PVA aqueous solution (~0.1 wt %) to the Ti precursor. As seen in Appendix A, the order of addition had great influence on the stability of the sol.

### 4.2. Powder and Film Preparation

Powder samples were obtained by drying the sol at 80 °C for several hours in a Pt crucible. The citrate method also required further drying at 120 °C to obtain a porous gel. The resulting powders were crushed and calcined at 250–800 °C in alumina crucibles and ambient atmosphere for 2 h. Samples with little (<1 mm height) or much (~5 mm height) crushed gel were heat treated simultaneously, and compared.

Thin films were prepared by spin-coating the sol onto 1 × 1 ${\mathrm{cm}}^{2}$ single crystal substrates of SrTi$O_{3}$ (STO) (100 oriented, Crystal GmbH, Berlin, Germany, Part No. 1STO 101E) and piranha etched platinized silicon (${\mathrm{Si}100|\mathrm{TiO}}_{2}|\mathrm{Pt}111$, MEMS Exchange, Reston, VA, USA). Nitrate sol samples were only deposited on STO substrates, and wetting during spin coating was enhanced by PVA addition to the sol. To further increase wetting of the platinized Si substrates, the substrates were heated to 200 °C on a hot plate, covered with sol, and cooled to room temperature. This led to a thick gel coating, which was rinsed off with water before the substrate again was covered with sol at room temperature and left resting for 5 min. Again, the sol/gel was rinsed off with water, and the subsequent addition of two drops of sol followed by promptly spin coating, gave good wetting with homogeneous thickness, as evident by a uniform iridescence color of both wet and dry films. The films were spin coated at 3000 rpm for 30 s, and then dried on a hot plate at 200 °C for 5 min. In order to ensure that secondary phases observed in thin film samples were not due to faulty sols, small batches of powders calcined at 800 °C were prepared from all sol batches. All of these were phase pure, demonstrating that the secondary phases were not a result of stoichiometry.

Films were pyrolized for 5 min in oxygen by rapid thermal processing (RTP), ramping at 40 °C/s to various temperatures (Jipelec Jetfirst 200 mm, Semco Technologies, Montpellier, France). A SiC coated graphite susceptor and lid surrounded the samples in order to protect the chamber from evolved gases. Spin coating and pyrolysis were repeated for each layer, in total 5 times. Finally, the samples were annealed at various temperatures for 10 min by RTP. During annealing, sacrificial powder of BNT was added to the susceptor in order to increase the vapor pressure of volatile Na and Bi, and thereby decrease the evaporative loss of these species.

### 4.3. Characterization

The phase purity and crystallinity were characterized by XRD (Bruker D8 Advance DaVinci, Karlsruhe, Germany), powders in Bragg-Brentano setup with a variable slit size, and films using grazing incidence setup with 2° incidence angle. All samples were measured with equal scan conditions, and no data manipulations other than vertical offset were applied. Rietveld refinements were carried out in Bruker AXS Topas version 5. Fundamental parameters peak shape was employed, with starting point in R3c reference unit cell parameters [30]. Background signal was refined for the most crystalline samples, and fixed to the same value for the remaining samples, where only unit cell parameters and crystallite size were refined. Powder samples were refined by the 2θ range 20°–75°, and films by 21°–49°.

Thermogravimetric analysis combined with mass spectroscopy (TGA-MS, Netzsch STA 449 C and Netzsch QMS 403 C, Selb, Germany) was conducted in synthetic air (20% $O_{2}$, 80% $N_{2}$) and argon on dried sols, heating and cooling at 10 °C/min. Scanning electron images were captured on a field emission gun SEM (Zeiss Ultra 55, Limited Edition, Oberkochen, Germany). Fourier transform infrared spectroscopy (FTIR) was done in vacuum by the attenuated total reflectance (ATR) method (Bruker Vertex 80v) on the ground calcined powders.

## 5. Conclusions

Two aqueous-based synthesis routes to BNT materials were developed in this work. Preparations of phase pure BNT thin films and powders were demonstrated by the use of the citrate method. The thermal decomposition of the gels obtained from the aqueous sol and formation of BNT were studied in detail by thermal analysis, FTIR spectroscopy, X-ray diffraction and electron microscopy.

The main challenge during the thermal processing of the materials was the reduction of ${\mathrm{Bi}}^{3+}$ to metallic bismuth during decomposition of the precursor gel, causing segregation of Bi and subsequent formation of a pyrochlore secondary phase. The parameters governing this effect were investigated, resulting in good control of the process yielding phase pure BNT thin films and powders. Parallels were drawn to similar synthesis routes of related materials, showing that the insight gained here may also be of value to these methods. The synthesis route was demonstrated to possess ideal properties for chemical solution deposition of BNT thin films, and is suitable for both further modifications of the composition of the films and up-scaling for industrial applications.

## Figures and Tables

**Figure A1:**
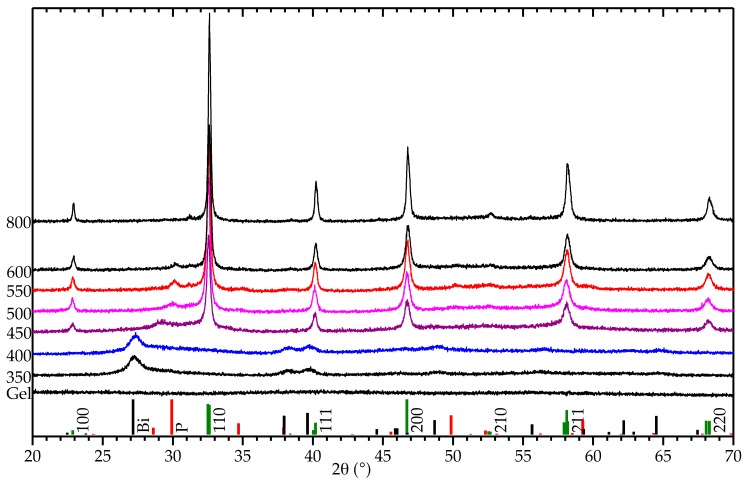
XRD patterns of calcined powders from nitrate method gel. All samples were calcined for 2 h in ambient atmosphere. Calcination temperatures are given to the left, and reference diffraction patterns for BNT [30], metallic bismuth, and pyrochlore [31] in the bottom.

**Figure A2:**
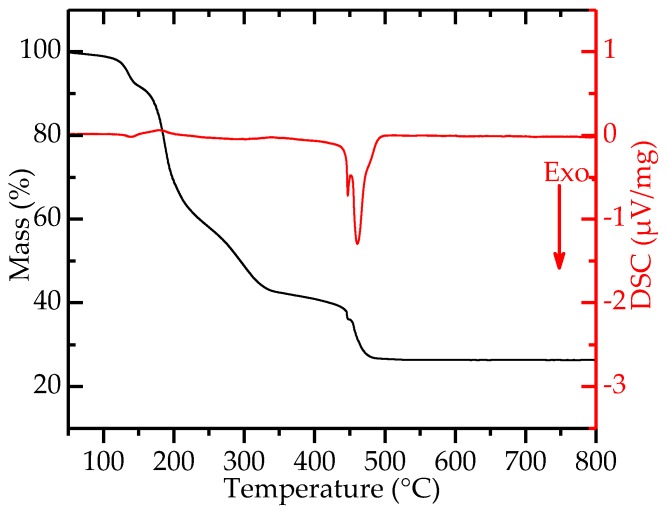
TGA measurement of BNT gel from nitrate method (10 °C/min ramp rate, synthetic air flow).

## Appendix A. The Nitrate Method

### Appendix A.1. Synthesis by the Nitrate Method

XRD patterns of samples prepared from the nitrate method gel by calcination at various temperatures for 2 h in air are shown in Figure A1. Phase pure powders were achieved, and crystallization of BNT perovskite phase occurred already at 450 °C. However, the reduction of bismuth at 300–400 °C is more pronounced with sharper diffraction lines, signifying larger metallic Bi crystallites, showing that the oxidizing properties of ${\mathrm{NO}}_{3}^{-}$ does not influence the reduction of Bi. Remnants of the bismuth phase formed early in decomposition can be seen as a diffraction line from a secondary phase peak at approximately 30° even after calcination at 500–600 °C.

TGA measurement of the nitrate method gel is shown in Figure A2. In accordance with the XRD data, the mass loss terminated at approximately 450 °C, 100 °C earlier than for the citrate method.

Due to inferior stability and pH of the nitrate method sol compared to the citrate method, fabrication of thin films was not investigated thoroughly. Pyrolysis at 500 °C and annealing at 700 °C yielded significant amounts of pyrochlore secondary phase, and additional annealing only increased the pyrochlore to BNT ratio.

While phase pure powder samples could be prepared from the nitrate sol, successful films could not be achieved, and the properties of this sol are significantly inferior to those of the citrate method sol. First, the required low pH of the system represents a hazard during handling, and could potentially limit the feasibility of use with pH sensitive manufacturing equipment or chemical additions such as barium to the sol. Second, while left in storage, the sol developed NO/${\mathrm{NO}}_{2}$ gases, identifiable by yellow coloration and smell, which also led to increased pressure in the containers, again posing a safety hazard. Finally, the nitrate sol was only stable for a couple of days, limiting the practical application of the sol, and resulting in potentially uncontrolled homogeneity and reproducibility.

Some comments should be dedicated to the works of Xu et al. [24,25,26,27], who prepared BNT-BT by a method very similar to the nitrate method reported here. They employed tetrabutyl titanate instead of Ti-isopropoxide, but citric acid was used as complexing agent and the developed n-butanol was separated from the sol. Therefore, the resulting complex and overall Ti-precursor system should be identical to ours. They adjusted pH to neutral by addition of ${\text{NH}}_{4}\text{OH}$ before adding $\mathrm{Bi}{\left({\mathrm{NO}}_{3}\right)}_{3}\cdot {5H}_{2}O$. Then, the slurry was stirred for 1 h at 80 °C. In the present work, this procedure did not result in a precipitate free solution, and only when pH was kept below 1 hydrolysis and precipitation of bismuth was avoided

### Appendix A.2. Stability of the Sols

The Bi-ethanolamine precursor (1 M) showed no ageing effects after three months, and it has been reported to be stable for several months [28]. Mixing the Ti and Bi precursors without adding ${\text{NH}}_{4}\text{OH}$ caused a white precipitation, likely due to too low pH for the Bi precursor, but the precipitate re-dissolved upon addition of ${\text{NH}}_{4}\text{OH}$. The final sols containing NaOH, Ti-precursor, Bi-precursor and ${\text{NH}}_{4}\text{OH}$ (citrate method) was stable for months. Drying the sol resulted in gradually increasing viscosity, and finally a brittle sponge-like structure, as gas bubbles were trapped, and the sol turned into solid gel. The gel was homogeneous and amorphous, as shown by XRD in Figure 1.

Obtaining a stable sol proved challenging for the nitrate method. $\mathrm{Bi}{\left({\mathrm{NO}}_{3}\right)}_{3}\cdot {5H}_{2}O$ was easily dissolved in water, but with pH above approximately 1–2, a white precipitate was immediately formed on the nitrate surface. For this reason, ${\mathrm{HNO}}_{3}$ corresponding to 1 M final concentration and PVA was added to the Ti precursor in advance of adding the cation nitrates. If the sol was to be diluted, distilled water had to be added and thoroughly mixed in advance of dissolving bismuth nitrate, as local pH values above 2 during dilution would cause precipitation which would not re-dissolve. Furthermore, as long as the Ti precursor was concentrated enough (0.6 M in Ti, 1.8 M in citric acid), the low pH from the citric acid alone was enough to properly dissolve$\;\mathrm{Bi}{\left({\mathrm{NO}}_{3}\right)}_{3}\cdot {5H}_{2}O$, however, these sols were not stable for a long time. Heating and stirring the solutions only increased the level of precipitation, indicating that the sols were only stable due to slow kinetics. Properly prepared nitrate method sols were stable for 1–2 days. As with the citrate method, drying the sol produced homogeneous and amorphous gels, and spin coating could also be carried out within the stability timeframe.

## Appendix B. Rietveld Refinements

**Table A1:** Refined structural parameters for BNT phase in relevant samples. An asterisk (*) marks samples that are phase pure. Refinement of XRD patterns from films on SiPt required simultaneous refinement of Pt to allow BNT peaks to properly relax. Note that refinement of patterns from film samples results in too small crystallite sizes due to the inherent broad peaks obtained using grazing incidence XRD set up.

Powders				Films on STO				Films on SiPt			
Heat Treatment (°C)	a (Å)	c (Å)	Cryst. Size (nm)	Heat Treatment (°C)	a (Å)	c (Å)	Cryst. Size (nm)	Heat Treatment (°C)	a (Å)	c (Å)	Cryst. Size (nm)
Reference: R3c [30]	5.49	13.50		Stoichiometric				Stoichiometric			
				450	5.50	13.52	13	450	5.50	13.49	8
Citrate				500	5.51	13.50	10	500	5.50	13.48	10
500 Little *	5.50	13.51	20	550 *	5.51	13.49	11	550	5.49	13.51	13
550 Little *	5.50	13.51	23	400 + 700	5.50	13.49	16	400 + 700	5.49	13.44	19
600 Little *	5.50	13.52	22	450 + 700	5.50	13.48	17	450 + 700	5.49	13.45	16
800 Little *	5.49	13.48	52	500 + 700 *	5.50	13.47	16	500 + 700	5.49	13.45	16
500 much	5.51	13.49	12	550 + 700 *	5.49	13.51	19	550 + 700 *	5.49	13.49	16
550 Much	5.50	13.52	17	400 + 800	5.49	13.47	18	400 + 800	5.48	13.43	13
600 Much	5.50	13.51	18	550 + 800 *	5.49	13.49	22	550 + 800	5.48	13.46	15
800 Much	5.48	13.46	54	400 + 900	5.49	13.44	22	400 + 900	No BNT		
Nitrate				550 + 900 *	5.49	13.41	30	550 + 900			
450	5.49	13.50	22	Na excess				Na excess			
500	5.49	13.51	21	450	5.52	13.51	6	450	5.50	13.48	8
550	5.49	13.50	26	500 *	5.50	13.51	13	500	5.49	13.51	13
600	5.49	13.49	28	450 + 700	5.49	13.51	20	450 + 700	5.48	13.50	18
800 *	5.49	13.47	44	500 + 700 *	5.49	13.50	19	500 + 700 *	5.48	13.49	20
